# Circadian gating of neuronal functionality: a basis for iterative metaplasticity^1^

**DOI:** 10.3389/fnsys.2014.00164

**Published:** 2014-09-19

**Authors:** Rajashekar Iyer, Tongfei A. Wang, Martha U. Gillette

**Affiliations:** ^1^Department of Cell and Developmental Biology, University of Illinois at Urbana-ChampaignUrbana, IL, USA; ^2^Department of Molecular and Integrative Physiology, University of Illinois at Urbana-ChampaignUrbana, IL, USA

**Keywords:** circadian rhythms, gating, plasticity, iterative metaplasticity, hippocampus, suprachiasmatic nucleus, glutamatergic, signaling

## Abstract

Brain plasticity, the ability of the nervous system to encode experience, is a modulatory process leading to long-lasting structural and functional changes. Salient experiences induce plastic changes in neurons of the hippocampus, the basis of memory formation and recall. In the suprachiasmatic nucleus (SCN), the central circadian (~24-h) clock, experience with light at night induces changes in neuronal state, leading to *circadian plasticity*. The SCN's endogenous ~24-h time-generator comprises a dynamic series of functional states, which gate plastic responses. This restricts light-induced alteration in SCN state-dynamics and outputs to the nighttime. Endogenously generated circadian oscillators coordinate the cyclic states of excitability and intracellular signaling molecules that prime SCN receptivity to plasticity signals, generating nightly windows of susceptibility. We propose that this constitutes a paradigm of ~24-h *iterative metaplasticity*, the repeated, patterned occurrence of susceptibility to induction of neuronal plasticity. We detail effectors permissive for the cyclic susceptibility to plasticity. We consider similarities of intracellular and membrane mechanisms underlying plasticity in SCN circadian plasticity and in hippocampal long-term potentiation (LTP). The emerging prominence of the hippocampal circadian clock points to iterative metaplasticity in that tissue as well. Exploring these links holds great promise for understanding circadian shaping of synaptic plasticity, learning, and memory.

## Introduction

The ability of salient stimuli to induce persistent changes in the structure and function of neurons is a fundamental modulatory process that confers the ability to modify physiology and behavior, learning from experience (Markham and Greenough, 2004). Although observed throughout the brain, plasticity is best studied in the hippocampus, a site critical to establishing and recalling new memories. Cellular and molecular mechanisms by which stimuli generate synaptic changes in hippocampal sub-regions fall into two classes: those that cause long-term potentiation (LTP) vs. long-term depression (LTD). Plastic changes in the hippocampus develop along linear timelines, initiated by the stimulus, followed by multiple sequential steps that are necessary to establish long-term, persistent functional changes.

Among the most salient stimuli for life on Earth are the alternating environmental states of day and night. The cycle of day and night partitions the availability of energy, both thermal and nutrient. Alternating activity vs. rest behaviors align with these environmental states. Internally, day-night changes in metabolism, physiology, and behavior are organized by an endogenous timekeeping system that oscillates with a circadian (*circa*, about, and *dian*, a day) period.

The multitude of interacting elements that generate and coordinate circadian rhythms throughout the body constitute the circadian timing system. Endogenous circadian rhythms are fundamental properties of all cells. They emerge from self-regenerative oscillations in transcription-translation of core timing (clock) genes, gene products, and their post-translational modification and in cellular metabolism of reduction-oxidation (redox) states. Cellular circadian rhythms of various tissues, e.g., heart, lung, blood, and brain, are synchronized by the hypothalamic suprachiasmatic nucleus (SCN) (Lehman et al., 1987; Ralph et al., 1990; Yoo et al., 2004). The SCN coordinates the myriad body clocks via diverse output signals and adjusts its circadian state via inputs that communicate desynchronization with environmental and internal conditions. Light-stimulated resetting of SCN circadian phase is a form of neuronal plasticity, *circadian plasticity*, mediated by cellular processes similar to those that cause long-term changes in hippocampal state, but susceptibility is temporally restricted to night.

As understanding of mechanisms that lead to memory formation in the hippocampus has grown, questions have arisen about how such plasticity is regulated to prevent network hyper-stimulation, saturation, and impaired recall and cognition. This led to the prediction and substantiation of metaplastic regulatory mechanisms. *Metaplasticity* is a persistent change in the state of synapses or neurons due to past activity or modulation that alters responses to subsequent plasticity-inducing stimuli. Consequently, responses may be altered in amplitude or duration compared with the basal response (Abraham and Bear, 1996). Metaplastic modulation can be local, involving single or nearby synapses, or extend throughout the neuron or network (Hulme et al., 2014). Behavioral expression of metaplasticity includes responses to environmental stimuli (enrichment or stress), developmental deprivation (visual or tactile stimulation), and changes due to associative learning (Abraham, 2008).

In this review, we consider the commonality of mediators of plasticity effectors and mechanisms between LTP in the hippocampus and circadian plasticity in the SCN. Once initiated, these feed-forward processes involving excitability and intracellular networks transform these systems into new, persistent states. On a higher-order level, the ability of the SCN to express plasticity-induced state changes is dependent upon the context that is modulated by the cycling circadian clock and the associated state changes in SCN neurons. This susceptibility to light-induced plasticity returns each night, as the circadian clock repeats its daily cycle, a phenomenon we term *iterative metaplasticity*. We propose that the hippocampal circadian clock similarly modulates the potential for LTP and other forms of synaptic plasticity by iterative metaplasticity.

## Why are there internal circadian systems?

The internal milieu is not resting in a basal state when stimuli are encountered. Rather, the internal environment—from the level of cells to tissues, organs, and brain and body systems–oscillates with a major regular periodicity near 24 h, as well as various ultradian rhythms. Consequently, patterning of behavioral outputs changes significantly over this period, so that some behaviors occur in the day, others at night, and some are expressed at dawn and dusk.

Daily internal oscillations are a result of adaptation to a major environmental variable over the course of evolution: the ever-changing cycle of day and night generated by the Earth's rotation on its axis. Early organisms that optimized cycles of behavior to adapt to these changes would have held a competitive advantage (Ouyang et al., 1998; Woelfle et al., 2004; Gerstner, 2012). Indeed, selection for mechanisms that internalized timekeeping embedded circadian clocks within genomes and basal metabolism (Lowrey and Takahashi, 2011; O'Neill and Reddy, 2011; O'Neill et al., 2011; Gillette and Wang, 2014). This encoded the patterning of environmental state within genes of some prokaryotes and all eukaryotes, enabling them to organize day vs. nighttime processes internally and predict these environmental state changes.

Rhythmic behaviors that anticipate rather than merely react to environmental changes would offer significant benefits. Functional and physiological changes can be initiated *in advance* of environmental changes, optimizing behavior and aligning it more closely to daily environmental cycles. Further, because day-night durations undergo seasonal variations, an anticipatory, endogenous rhythm generator would adapt and entrain to changing ratios of light: dark periods, generating seasonal plasticity in behaviors (Bartness et al., 1993; Ebling et al., 1995; Nuesslein-Hildesheim et al., 2000; Meijer et al., 2010).

Anticipatory, near-24-h *circadian rhythms* are generated by molecular and cellular processes, and result in appropriately timed cycles of physiology, metabolism, and behavior. They persist in constant darkness. The endogenous period of circadian clocks under these aperiodic conditions is close-to, but not exactly, 24-h (King and Takahashi, 2000; Okamura et al., 2002). Consequently, *free-running* rhythms drift out of correspondence with day and night of the solar cycle. They re-adjust in response to light signals that occur during the subjective night of circadian clock processes. Thus, when animals are exposed to regular 24-h cycles of light and dark or light presented briefly at regular 24-h intervals, the rhythms undergo *entrainment*, aligning with the time-schedule dictated by light (Golombek and Rosenstein, 2010). Because light can communicate environmental timing to entrain the circadian system, it is a *zeitgeiber* (time-giver).

## Circadian rhythms are generated by molecular and metabolic oscillators

How do molecules and metabolism generate self-sustaining near 24-h oscillators? The endogenous timing mechanism consists of a transcription-translation oscillator (TTO) with negative feedback (Lowrey and Takahashi, 2011) that interacts with a reduction-oxidation oscillator (RXO) (Gillette and Wang, 2014). In the TTO, heterodimers of positive transcription factors CLOCK/BMAL (or CLOCK/NPAS2 in some brain regions) bind to E-box motifs in the promoters of *Per 1/2/3*, *Cry 1/2*, and *Rev-Erb*α, activating transcription. The protein products undergo complex post-translational modifications. Under appropriate conditions, PER and CRY proteins heterodimerize, translocate to the nucleus, and repress transcriptional activation by CLOCK/BMAL. An additional regulatory feedback loop involves the negative regulator, REV-ERBα (a nuclear heme receptor) and the positive regulator, retinoic acid-related orphan receptor (ROR) (Lowrey and Takahashi, 2004; Yin et al., 2007, 2010). These antagonistic regulators compete for binding to ROR elements (ROREs) within the promoters of *Bmal1* and *CLOCK*. REV-ERBα also can modulate transcription by binding to ROREs in the *Per* and *Cry* promoters. Transcription-factor binding is a dynamic process, which permits regulation based on relative amounts and states. Rates of synthesis and proteasomal degradation of clock proteins are important to rhythm generation (Gillette and Mitchell, 2002; Nitabach et al., 2002; Lundkvist et al., 2005; Golombek and Rosenstein, 2010; Van Ooijen et al., 2011). When PER and CRY are ubiquitinated and degraded, the cycle of *Per* and *Cry* transcription-translation repeats. The TTO takes ~24-h to complete one cycle, as does an accessory loop comprising the transcription factor REV-ERBα. REV-ERBα binds to ROREs in promoter regions of the *Clock* and *Bmal1* genes, initiating their transcription.

The RXO emerges from robust, near-24-h rhythms of cellular metabolic state. Redox state oscillates in SCN samples *ex vivo*, as well as brain slices *in vitro*. This RXO modulates the membrane potential (V_m_) of SCN neurons and thus the state of excitability (see further). Drivers of the RXO are presently unknown, however, redox oscillations are wide spread in circadian systems (O'Neill and Reddy, 2011; O'Neill et al., 2011). Transcriptional regulation via REV-ERBα, an endogenous heme receptor, is exquisitely sensitive to cellular redox state. This is one of several nodes of interaction between the TTO and the RXO (Gillette and Wang, 2014). The interacting oscillators generate rhythms in the synthesis of key cellular proteins, firing rate of neurons, and release of neuropeptides (Hatcher et al., 2008; Wang et al., 2012). These clock-controlled rhythms can have distinct phase-relationships, patterns, and amplitudes. Time-of-day restrictions on susceptibility to phase-resetting signals emerge from the complexity of theses dynamic systems.

## Circadian oscillation in SCN excitability

The daily rhythm of electrical activity in SCN neurons is essential for the central pacemaker to synchronize circadian clocks throughout the body to each other and to changing environmental time cues (Brown and Piggins, 2007; Colwell, 2011). SCN neurons exhibit a daily fluctuation of spontaneous action potentials (SAP), with higher frequency during the daytime than the night. The SAPs are autonomously generated by the SCN (Brown and Piggins, 2007), and their patterned behavior can be detected both *in vivo* and *in vitro*, by single- or multi-unit recording (Inouye and Kawamura, 1979; Green and Gillette, 1982; Welsh et al., 1995). Dissection of underlying ionic mechanisms by patch-clamp recordings of membrane properties of SCN neurons from mouse (Belle et al., 2009) and rat (Wang et al., 2012) reveals at least three ionic factors, K^+^ and Ca^2+^ currents and [Ca^2+^_i_], underlie rhythmic oscillating membrane potential (V_m_) (Belle et al., 2009). These ionic features represent three functional categories (Colwell, 2011): (1) currents *providing* the excitatory drive that elevates V_m_ to the threshold of action-potential generation; (2) currents *responding* to the excitatory drive and generating action potentials; and (3) currents contributing to the nightly silencing of firing through hyperpolarization of the membrane. Modulation of these currents could be on the levels of channel expression, localization, post-translational modification of conductance, and/or gating properties. Circadian oscillation in V_m_ is necessary for timekeeping. Electrical silencing of pacemaker neurons in *Drosophila* by genetic manipulation of K^+^ channels stops the free-running circadian clock, resulting in arrhythmic behavior (Nitabach et al., 2002).

While links between oscillations in V_m_ and circadian plasticity have not been fully established, recent studies suggest important roles for the neuropeptides released in the SCN. Their circadian rhythm is driven by oscillation in V_m_, which fine-tunes the neuronal responses to input signals. Among the more than 200 neuropeptides identified in SCN, vasoactive intestinal peptide (VIP) is the most characterized and well studied. VIP is an essential neuropeptide for the synchrony of the brain clock (Aton et al., 2005). VIP binds to a G-protein-coupled receptor (VIP•R 2), activating cAMP and PKA pathways (Hao et al., 2006). cAMP is essential for the maintenance of intrinsic circadian rhythmicity; it is also important in information processing within the brain clock (Hastings et al., 2008). cAMP was the first confirmed non-transcriptional cytosolic oscillator in SCN neurons (O'Neill et al., 2008), followed by Ca^2+^ (Harrisingh et al., 2007), PKC (Robles et al., 2010), small G protein (Brancaccio et al., 2013), and other small molecules (Dodd et al., 2007).

Beyond VIP, numerous neuropeptides have been found to be important to regulating clock function. Examples include the following. **(1)** Mice lacking arginine vasopressin (AVP) receptors are more resistant to jet-lag and their recovery period is more rapid than wildtypes (Yamaguchi et al., 2013). This feature results from the looser coupling between cells in AVP receptor-deficient SCN, which maintains the basic circadian rhythm under steady state, but is more responsive to *zeitgebers* for extreme phase-shift. **(2)** Gastrin-releasing peptide (GRP) is another major neuropeptide in the brain clock. Activation of GRP-receptors produces long-lasting excitation in SCN neurons (Gamble et al., 2007). This response is stronger in subjective night than daytime. The underlying mechanism involves inhibition of resting K^+^ current and subsequent membrane depolarization (Gamble et al., 2011). **(3)** Pituitary adenylate cyclase-activating peptide (PACAP) is present in retinohypothalamic tract (RHT) terminals that invest SCN tissue. PACAP alters the excitability of SCN neurons in a bi-modal manner: it suppresses the discharge of some of the neurons via VIP•R2 receptors, while excites others via VIP•R1 receptors (Reed et al., 2002). PACAP also modulates light-induced phase-shift in complex ways: it facilitates phase-delay but attenuates phase-advance; the underlying mechanism has yet to be revealed (Chen et al., 1999).

The SCN releases these, and many other neuropeptides, with levels of release following a circadian pattern (Hatcher et al., 2008). It is thought that these neuropeptides also play a role in synchronizing clocks in other brain regions by carrying time-of-day information from the SCN (Lee et al., 2010). It follows that neuropeptides may contribute to clock-based metaplasticity.

## The many brain connections of the SCN

The SCN has direct and indirect connections with many brain sites. Recent assessments identify 35 brain regions projecting directly to the SCN, and if multi-synaptic inputs are included, this number increases to 85 projecting regions (Morin et al., 2006; Morin, 2013). Input from the retina via the retinohypothalamic tract (RHT), the thalamic intergeniculate nucleus via the geniculohypothalamic tract (GHT) and the median raphae nucleus are considered critical to the “circadian visual system” (Morin and Allen, 2006). A study of the various primary and secondary inputs conducted using retrograde tracers (Krout et al., 2002) found many sources for SCN afferents, including regions in the hippocampal formation. Many brain regions are targets for efferent projections from the SCN (Abrahamson and Moore, 2001; Morin, 2013). Of particular interest is the indirect connection of the SCN, via the dorsomedial hypothalamus, to the locus coeruleus (Aston-Jones et al., 2001; Markov and Goldman, 2006), a region known to mediate cortical and hippocampal activation (Berridge and Foote, 1991).

## Circadian clocks in the brain

The SCN is acknowledged to be *the* central circadian clock in mammals (Lehman et al., 1987; Ralph et al., 1990). The SCN maintains its autonomous circadian rhythm when surgically isolated from the rest of the brain *in vivo* (Inouye and Kawamura, 1979) and *in vitro*, where synchronized near-24-h rhythms of electrical activity, metabolism, and clock-gene expression persist (Schwartz et al., 1980; Gillette and Prosser, 1988; Prosser et al., 1989; Yamazaki et al., 1998; Wang et al., 2012). Whereas all cells of the body possess intrinsic clocks, in the absence of the SCN the myriad cellular TTOs drift out of phase with one another. This was demonstrated elegantly in a mouse bearing a transgene reporter, PER 2::LUCIFERASE, where circadian rhythms in this clock protein are expressed as bioluminescence and can be measured non-invasively in all cells and tissues (Yoo et al., 2004). When tissues were assessed *in vivo* after SCN lesion or cultured in isolation *in vitro*, individual cells from all tissues examined continued to exhibit circadian oscillations, but with a range of phases that appeared arrhythmic when averaged (Welsh et al., 1995; Yoo et al., 2004).

Whereas the rest-activity cycles of nocturnal and diurnal animals are in anti-phase, SCN electrical activity in nocturnal and diurnal animals alike peaks during the light phase (Fuller et al., 2006; Brown and Piggins, 2007). This indicates the switch in peak activity in other brain regions that drive the behavioral differences between nocturnal and diurnal animals lies downstream of the SCN, and affirms the autonomy of SCN electrical activity rhythms. Extra-SCN brain tissue, such as the paraventricular nucleus (PVN), drifts out of synchrony *in vitro* but rapidly re-synchronizes to the rhythm of the SCN in co-culture (Tousson and Meissl, 2004). Mediators of this inter-region synchronization are subjects of current study. Multiple modes of communication of phase have been identified, including the electrical communication via neuronal circuits, as well as via diffusible signals (Silver et al., 1996; Tousson and Meissl, 2004; Guo et al., 2005; Kalsbeek et al., 2006; Welsh et al., 2010). Thus, in each cell of the body an endogenous circadian clock controls the daily timing of cell-specific transcription, cell dynamics, and signaling, but it relies on information from the SCN for coordination.

In addition to its autonomous ~24-h rhythmicity, the SCN holds a privileged position among circadian clocks at the cell and systems levels in receiving direct information about the presence and intensity of environmental light from the retina via the RHT. It is then able to transmit this information to peripheral clocks. Intrinsically photoreceptive retinal ganglion cells (ipRGCs) act as photon-counters, marking the presence, duration, and intensity of light (Berson et al., 2002; Hattar et al., 2002). Their axons innervate the SCN and communicate the presence of light by releasing glutamate and the co-localized peptide, PACAP, onto the ventral SCN (Chen et al., 1999). The SCN integrates these signals and transmits information about the environmental light profile to all other cellular circadian clocks in the brain and body. The SCN also receives timing signals from other, less potent, *zeitgeibers* including locomotor activity, sleep/wake states and nutritional status (Stephan, 1989; Lamont et al., 2005; Fuller et al., 2006), resulting in feedback control of the circadian timing system.

Circadian rhythms of clock genes have been reported in several brain regions, including the prefrontal cortex, olfactory bulb, and hippocampus (Abe et al., 2002; Granados-Fuentes et al., 2006; Li et al., 2013). The hippocampus exhibits circadian oscillation in the expression of *Per2*, a hallmark of the TTO. The amplitude and persistence of LTP in the CA1 region varies in a circadian manner (Chaudhury and Colwell, 2002; Chaudhury et al., 2005). Mutations in *Per 2* that impair the circadian clock result in abnormal hippocampal LTP (Wang et al., 2009). This supports a necessary role for the circadian clock in permitting and enabling hippocampal plasticity.

## Circadian oscillation in susceptibility to light-induced plasticity

The SCN processes information about significant variations in availability of light or nutrients that necessitate adjustment of circadian timing. It dynamically responds to cues that communicate mismatch between internal and environmental time. Light, the signal of day-length, alters clock gene expression, as well as phases of the circadian oscillations in heart rate, ingestion, and wheel-running behavior.

**Figure 1:**
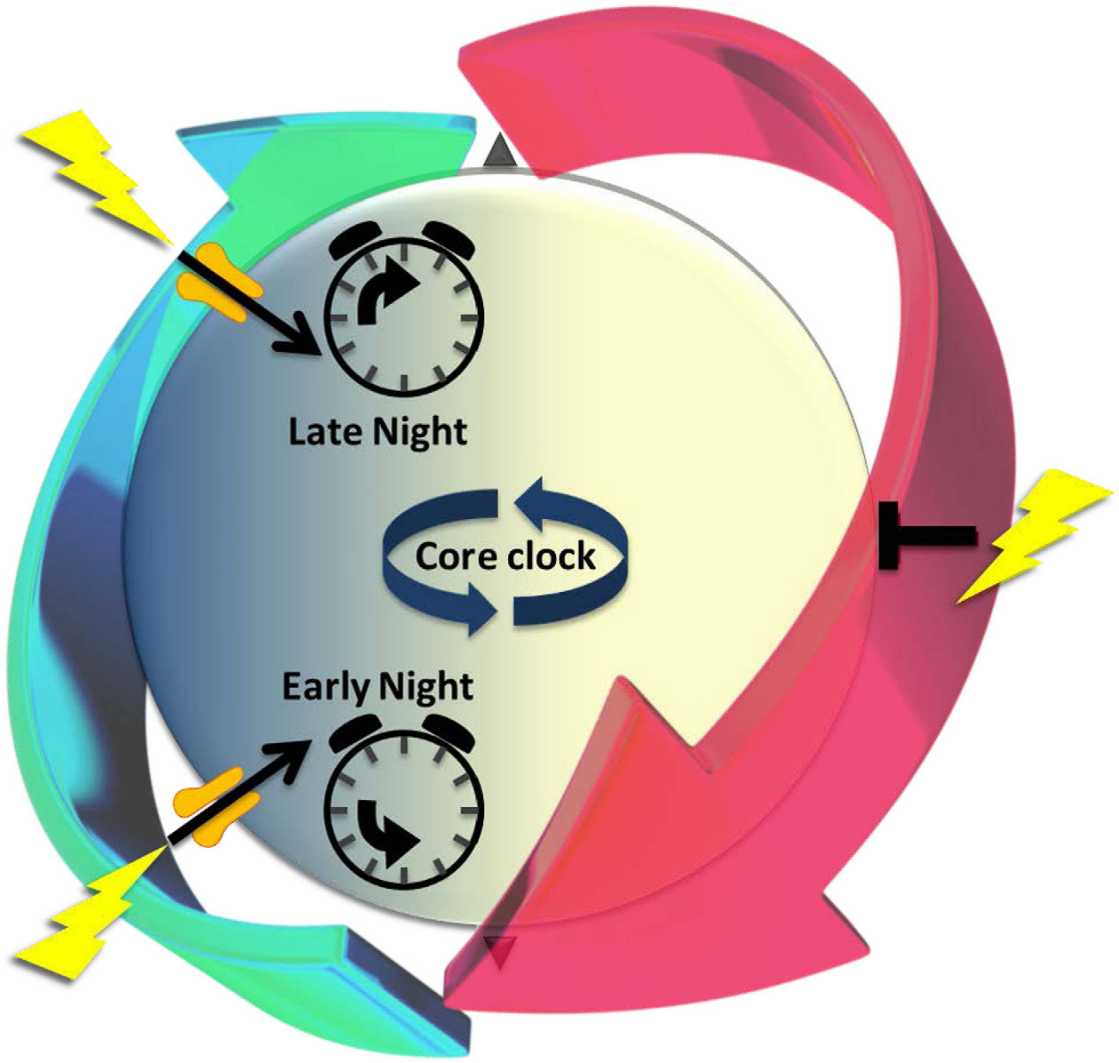
**The cyclic series of dynamic cellular states of the endogenous circadian clock is characterized by changing susceptibility that recurs every ~24 h to signals that alter clock state, a paradigm of *iterative metaplasticity***. The periodic recurrence of night and day provides an oscillatory environmental context for life on Earth. A near-24-h dynamic series of functional states organizes differential responses to light that depend upon time-of-day. Light-driven glutamatergic signals (lightning bolts) at night (blue) alter suprachiasmatic nucleus (SCN) state-dynamics and outputs; daytime (yellow) stimulation is without effect. Circadian timekeeping mechanisms generate these windows of susceptibility, poising the SCN to respond appropriately to a temporal error signal- in early night moving clock state back to an earlier time or in late night advancing it prematurely toward morning. These long-lasting changes in clock state express the hallmarks of neuronal plasticity. The gate to light-signaling is open transiently during nighttime (green arrow), but closed in daytime (red arrow). The gating mechanisms permissive for state changes are clock-driven, preceding the light signal. Thus, light-induced plasticity occurs only if the functional state of cells is permissive at that time. Underlying differences in susceptibility are cyclic states of excitability and intracellular signaling elements that prime SCN receptivity to plasticity signals. We propose that this gating of light-signaling responsiveness, which cycles over the night and day, is a paradigm of *iterative metaplasticity*, the repeated, anticipatory susceptibility to induction of neuronal plasticity.

The SCN has the unusual property of responding to light differently at different points in the circadian cycle (Figure 1). The phase of rhythms in clock gene expression, neuronal firing, and locomotor activity are unaffected by light in the daytime, but change significantly when light is encountered during nighttime. The response to nocturnal light is bifurcated (Ding et al., 1994). Light in the early night (the period after the normal light-off) signals a prolonged day; the clock responds by delaying its phase. In the late night (the period before lights-on), however, light signals an early dawn, advancing clock phase prematurely to a daytime state. The SCN generates such differential responses by selectively *gating* its susceptibility to inputs (see further). Gating is regulated at multiple levels, including neurotransmitter receptors and effectors of intracellular signaling pathways (Gillette and Mitchell, 2002; Golombek and Rosenstein, 2010).

Light is communicated to the SCN from the retina by glutamatergic neurotransmission from the RHT (Ding et al., 1994; Welsh et al., 2010). During subjective nighttime, glutamate or the agonist N-methyl D-aspartate (NMDA), are sufficient to activate light signaling *in vivo* (Colwell and Menaker, 1992; Vindlacheruvu et al., 1992; Gannon and Rea, 1993, 1994) or the SCN brain slice *in vitro* (Ding et al., 1994; Shirakawa and Moore, 1994). They mimic the effects of light: changing circadian phasing, inducing *c-fos* and other immediate early genes (Ebling, 1996; Guido et al., 1999) and the clock gene *Per 1* (Moriya et al., 2000). NMDA receptor antagonist application effectively blocks light-induced changes. In subjective daytime, glutamate exposure does not activate these signaling pathways, nor does it cause a change in phase.

**Figure 2:**
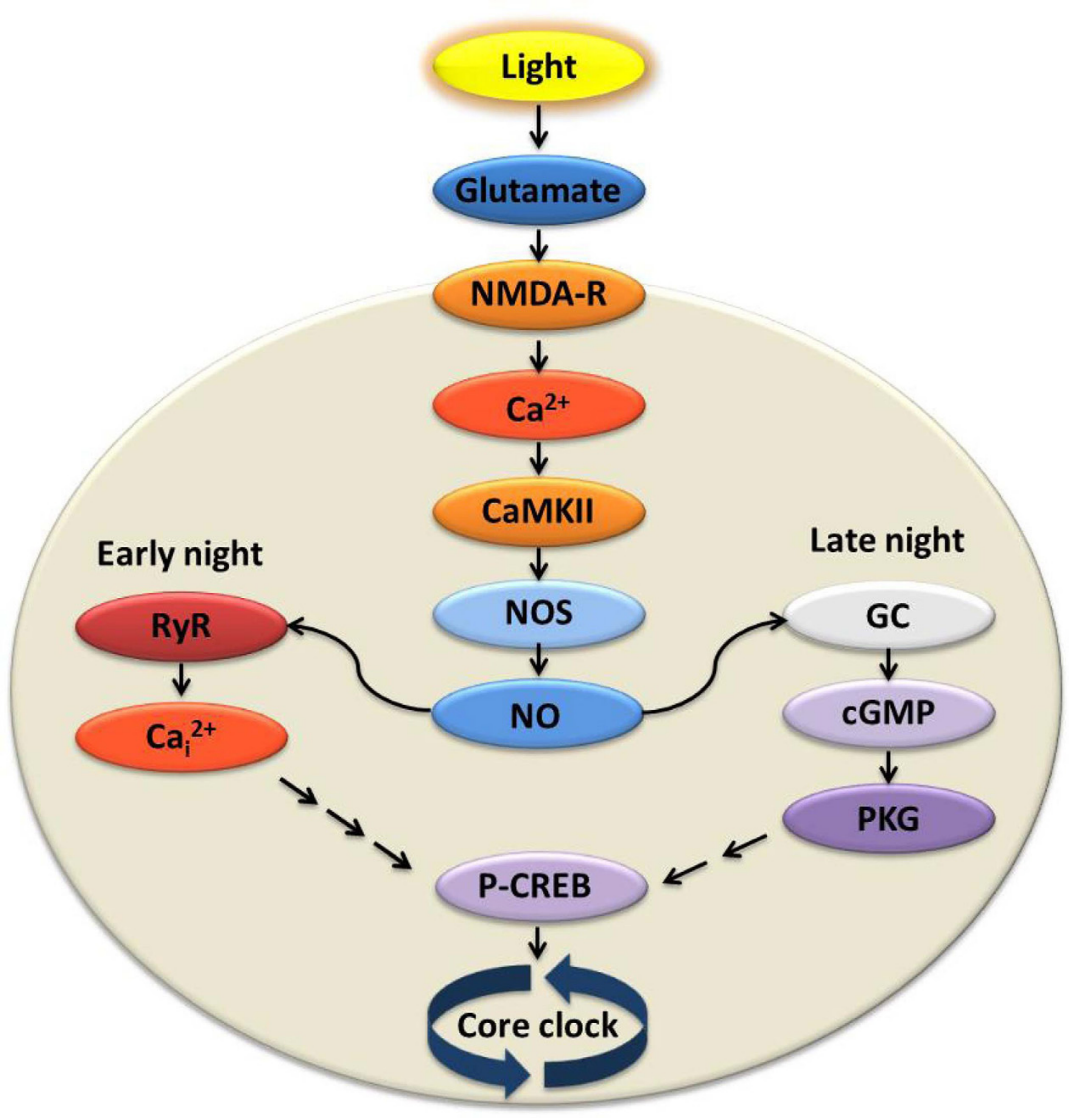
**Signal transduction at the suprachiasmatic nucleus (SCN) in response to light activates divergent pathways in early vs. late night**. Light experienced at night transmits signals via the retinohypothalamic tract (RHT) to the SCN causing glutamate release. Glutamatergic activation of the NMDA receptor is necessary and sufficient for initiating state changes, and leads to influx of extracellular Ca^2+^. Ca^2+^/calmodulin-dependent kinase II (CaMKII) and nitric oxide synthase (NOS) are activated, increasing levels of nitric oxide (NO). In the early night, the rise of NO activates ryanodine receptors (RyR) on the intracellular endoplasmic reticulum where Ca^2+^ is stored. Intracellular Ca^2+^ (Ca^2+^_i_) is released via the activated RyR and, through a mechanism yet to be elucidated, leads to phosphorylation of cAMP response element-binding protein (pCREB) and subsequent increased expression of clock genes. During the late night, however, NO activates guanylyl cyclase (GC), cGMP synthesis, and increased activity of cGMP-dependent protein kinase (PKG). Activation of this and other kinases again leads to increased pCREB and transcription of key clock genes. This simplified model includes only those elements necessary and sufficient to stimulate state changes similar in amplitude and timing to light-induced responses.

Initial steps of glutamate signaling include NMDA receptor (NMDA•R) activation, Ca^2+^ influx into cells in the SCN (Ding et al., 1998; Obrietan et al., 1998; Colwell, 2000, 2001), and stimulation of downstream kinases (Figure 2). Among the earliest changes is in Ca^2+^/calmodulin-dependent kinase Type II (CaMKII), which is activated by auto-phosphorylation. Inhibition of CaMKII in early night blocks light-induced phase delays and changes in *c-fos* and *Per 1/Per 2* expression in the SCN (Golombek and Ralph, 1995; Fukushima et al., 1997; Yokota et al., 2001). Active pCaMKII phosphorylates neuronal nitric oxide synthase (nNOS) (Agostino et al., 2004), triggering nitric oxide (NO) production. NOS activity is necessary for light-induced plasticity in the SCN (Ding et al., 1994, 1997; Melo et al., 1997), and the response to light or glutamate increases in the presence of an NO donor (Melo et al., 1997).

The light signaling pathway bifurcates in early vs. late night, downstream of NO (Figure 2). During the *early night*, glutamate-induced plasticity requires Ca^2+^-induced-Ca^2+^ release (CICR) from neuronal ryanodine receptors (RyRs). When RyRs are pharmacologically activated, the effects of light/glutamate in the early night are reproduced. The same agents have no effect in late night or daytime. Further, inhibition of RyRs blocks light-/glutamate-induced phase delay in the early night both *in vivo* and *in vitro*, but has no effect in late night or daytime (Ding et al., 1998).

During *late night*, on the other hand, NO activates guanylyl cyclase (GC), which increases cGMP levels. This in turn leads to the activation of cGMP-dependent kinase (PKG) (Weber et al., 1995; Ding et al., 1998; Tischkau et al., 2003b). Phase advance caused by light/glutamate in the late night is mimicked by cGMP analogs (Prosser et al., 1989) and blocked by pharmacological inhibition of PKG (Weber et al., 1995; Mathur et al., 1996; Ding et al., 1998; Ferreyra and Golombek, 2001). The same inhibition does not impact the phase delay induced in early night.

In both pathways, downstream from the steps described, there is transient and rapid phosphorylation of the Ca^2+^-cAMP response element-binding protein (CREB), leading to transcription of *Per 1*, as well as other CRE-mediated genes (Ginty et al., 1993; Ding et al., 1997; Gau et al., 2002). Phase advance during late night is blocked by antisense oligodeoxynucleotides (αODNs) of CRE sequences. These Ca^2+^/cAMP response element decoys (CRE-decoys) sequester pCREB and selectively block the advance in clock phase (Tischkau et al., 2003a).

Other factors downstream of glutamate-induced signaling in the SCN are the mitogen-activated protein kinases (MAPKs) and cAMP-dependent kinase (PKA). Both light-induced phase delays and advances are partially blocked by inhibitors of p44 MAPK/ERK1 and MAPK kinase (MAPKK/MEK) transcription (Obrietan et al., 1998; Tischkau et al., 2000; Butcher et al., 2002; Antoun et al., 2012). Light pulses during the subjective night induce activation of members of all three MAP kinase pathways: ERK, JNK, and p38 (Butcher et al., 2003; Pizzio et al., 2003). Signaling via p44/42 MAPK (ERK1/ERK2) is necessary for Ca^2+^-induced CRE-mediated gene transcription (Obrietan et al., 1998; Butcher et al., 2002; Antoun et al., 2012). PKA activation by light and glutamate has differential effects on SCN state at early *vs*. late night. Upon light or glutamate stimulation, PACAP and PKA enhance the amplitude of light-/glutamate-induced phase delay in early night, and diminish the effect in late night (Tischkau et al., 2000). These effects are like those of PACAP, which were described earlier.

Various regulators of light-induced plasticity affect the network properties of SCN neurons. Brain-derived neurotrophic factor (BDNF) is expressed in the SCN. The cognate receptor of BDNF, tropomyosin-related receptor kinase B (TrkB), is expressed in the fibers of the RHT (Allen and Earnest, 2005). BDNF expression and release are rapidly increased by neuronal activity, which regulates presynaptic release and direct activation of membrane channels (Rose et al., 2004; Blum and Konnerth, 2005). This enhanced expression and response to optic nerve stimulation suggest a role for BDNF in modulating photic input to the SCN (Allen and Earnest, 2005). BDNF- and TrkB-deficient mice exhibit a reduction in the phase-shifting effects of light on the circadian system (Liang et al., 2000). BDNF cannot shift the phase of the clock itself (Prosser et al., 2008; Mou et al., 2009), but interfering with BDNF signaling in the SCN blocks or strongly inhibits phase shifts induced by light and glutamate in the subjective night (Liang et al., 2000; Michel et al., 2006). SCN treatment with either BDNF or tissue-type plasminogen activator (tPA) permits light and/or glutamate to induce phase shifts in the daytime (Liang et al., 2000; Mou et al., 2009). BDNF thus contributes to *gating* of SCN responsiveness to state-changing signals, a concept we will explore in the next section. Gating of the light-response in the SCN is conferred by regulaton of glutamatergic synaptic transmission in the SCN (Kim et al., 2006).

Time-of-day-dependent neurotransmission via the inhibitory neurotransmitter gamma-amino butyric acid (GABA) profoundly affects SCN responses to light. GABA and its biosynthetic enzyme, glutamatic acid decarboxylate (GAD), are widely expressed in SCN neurons (Card and Moore, 1984; van den Pol, 1986; Okamura et al., 1989; Moore and Speh, 1993). Both GABA_A_and GABA_B_ receptors have been localized within the SCN by binding and functional studies (Francois-Bellan et al., 1989; Liou et al., 1990; Mason et al., 1991). Further, both GAD activity and GABA levels undergo circadian oscillations in the rat SCN (Aguilar-Roblero et al., 1993). Modulatory effects of GABA signaling on light-induced plasticity are complex, and consistent with a model wherein GABA_B_ receptors regulate photic signals via presynaptically modulating glutamatergic input from the RHT, whereas GABA_A_ receptors are proposed to act in the photic signal transduction cascade in local circuits downstream of glutamatergic inputs (Ralph and Menaker, 1989; Gillespie et al., 1997). GABAergic activation of GABA_B_ receptors diminishes release of labeled glutamate at the SCN in response to optic nerve stimulation (Liou et al., 1986). Consistent with this observation, GABA_B_ receptor agonists reduce light-induced phase delays in early night; an antagoinist of GABA_B_ receptors enhances this phase delay (Gillespie et al., 1997). With regard to light-effects in late night, microinjections of agonists of either GABA_A_and GABA_B_ receptor agonists into the SCN region significantly diminish phase-advances. Thus, GABAergic modulation of glutamatergic and downstream synapses contributes to gating and amplitude of the light-response in the SCN. Inhibition of the phase shift has been proposed to be mediated by the opposing effects of light and GABA on *Period* gene expression (Ehlen et al., 2008). Additionally, GABA modulates light-induced plasticity by inducing divergent responses in intracellular Ca^2+^ mobilization in SCN neurons (Irwin and Allen, 2009).

Although the complete signaling cascade from light to clock gene expression remains unresolved, the roles of these well-studied effectors downstream of light/glutamate provide insights into the most completely studied form of *circadian plasticity* in the SCN (Figure 2). The elucidation of the roles of several of these effectors in SCN plasticity, including pCREB and RyRs, was influenced by their role in plasticity signaling in the hippocampus (Ding et al., 1994, 1997, 1998).

## Circadian gating of clock function: iterative metaplasticity

Because SCN responsiveness to light-induced state changes waxes and wanes with each daily circadian cycle, states of sensitivity to plastic change repeat each night, when light has the potential to interrupt the anticipated darkness. Thus, the nocturnal state of SCN activity alters the magnitude and/or duration of plastic events compared with the daytime state. Dynamic linkage of temporal history and state with current responsiveness defines *metaplasticity* (Abraham and Bear, 1996). At night, the SCN responds to light as an error signal–it infers a mismatch between the timing of the clock and the environmental light cycle. The SCN must be able to distinguish *nocturnal* light as an error signal to respond with temporally appropriate phase-resetting. The restricted, time-of-day dependence of susceptibility of the SCN to light establishes conditions for the *iterative metaplastic state*.

During the night, the manner in which phase is affected varies depending on proximity of the signal to the subjective dusk or dawn encoded in the circadian clock. Dynamic *gating* of inputs to the SCN ensures that the same input (light) has restricted and differential effects on output that depend on the time of day during stimulation. Gating is achieved by clock-driven rhythms in V_m_, expression of plasticity modulators, and expression/receptiveness of intracellular effectors of plasticity. This implies the presence of a reciprocal relationship wherein various inputs alter timekeeping elements and induce plasticity, and their oscillations are in turn controlled by the circadian clock. Circadian control of input allows the clock to prime SCN neurons in anticipation of the activation of specific signaling cascades at specific times of the day. Key signaling elements that are potential targets of metaplastic regulation follow.

### Membrane excitability

The proposed model is that SCN neurons are highly electrically active during daytime, preventing additional excitatory input from the RHT from inducing further changes. At night, however, the neurons are hyperpolarized, thus excitatory input from the RHT elicits significant responses. Supporting this hypothesis, up-regulation of K^+^ currents and the consequential hyperpolarized status of V_m_ during nighttime are essential for gating of phase-shift responses. In excitable cells, V_m_ is mainly set by a class of two-pore domain K^+^ channels (K2P, TASK, and TREK channels) (Hallie, 2001). Gating of these channels is independent of voltage across the membrane; the current is termed leak or background current. K2P channels are encoded by the KCNK gene family. Both *Kcnk1* and *Kcnk2* are expressed in the SCN (Lein et al., 2007); in particular, *Kcnk1* exhibits a robust rhythm in the SCN (Panda et al., 2002). Interestingly, a functional study suggests that the K^+^ leak current is regulated by metabolic oscillation in SCN neurons, which provides a non-transcriptional pathway of the clockwork machinery to modulate the membrane excitability (Wang et al., 2012).

### Intracellular Ca^2+^

Membrane excitability influences neuronal plasticity through intracellular signal pathways where Ca^2+^ plays an essential role. SCN neurons exhibit spontaneous oscillation of intracellular Ca^2+^ (Ca^2+^_i_) concentration, with peak levels during the daytime (Ikeda et al., 2003). While Ca^2+^_i_ oscillation is not completely driven by membrane events, the action potential-induced opening of voltage-gated Ca^2+^ channels (L-type) is a proven source of Ca^2+^_i_ elevation during the daytime (Irwin and Allen, 2007).

### NMDA receptor

The SCN exhibits circadian expression of mRNAs for subunits of the NMDA receptor (NMDA•R) subunits ε3 and ζ1 in rats; mRNA levels are high during daytime and low during nighttime, whereas protein levels change in anti-phase. Expression of these mRNAs increases in response to light stimulation in the subjective night (Ishida et al., 1994). Both expression and phosphorylation of the ε 2/NR2B subunit of NMDA•R undergo circadian variation in hamsters. NR2B mRNA level is high from late day through early night, with the phosphorylated protein peaking in the late night (Wang et al., 2008). Changes in phosphorylation of NMDA•R subunits can correspond to changes in functional properties. An endogenous daily rhythm in the magnitude and duration of NMDA•R-induced Ca^2+^ transients in rat SCN neurons peaks during the night, as does the rhythm in NMDA•R-evoked currents (Pennartz et al., 2001). Thus, evidence indicates that peaks in mRNA abundance of certain subunits precede peaks in NMDA•R phosphorylation. NMDA function peaks in the night in rats, anti-phase to mRNA abundance. Together, these observations indicate that the clock *primes* the SCN to respond to photic signals in anticipation of encountering light signals at night.

### CaMKII

Calmodulin kinase II (CaMKII), which is activated in neurons by elevated Ca^2+^_i_, undergoes circadian changes in autophosphorylation state in hamsters, with peak abundance of pCaMKII occurring during subjective day. Total CaMKII levels in the SCN do not vary. Light pulses during the subjective night rapidly phosphorylate CaMKII, which is necessary for the signaling cascade resulting in circadian plasticity (Agostino et al., 2004).

### cAMP/PKA

In constant conditions, the rat SCN exhibits spontaneous oscillations in cAMP levels and cAMP-dependent protein kinase (PKA) activity. The endogenous levels of cAMP peak at the end of day and of night (Prosser and Gillette, 1991). While cAMP levels rise in response to light/glutamate stimulation, application of agonists to cAMP/PKA does not mimic the effect of light/glutamate. However, if cAMP/PKA is activated simultaneously with light/glutamate stimulation, resultant plasticity is enhanced in early night, and diminished in late night. Thus, the cAMP/PKA system toggles responses, changing the state of signaling pathways based on time of activation (Tischkau et al., 2000).

### cGMP/PKG

cGMP levels and cGMP-dependent protein kinase (PKG) activity also display spontaneous oscillations in the SCN. Tissue cGMP levels remain relatively constant throughout subjective day and night, with a sharp peak appearing at the time corresponding to end of night. PKG activity is markedly higher at end of subjective night compared to the end of subjective day and the middle of subjective night. Sensitivity of the SCN to light-induced increase in cGMP levels and PKG activity occurs just before these values peak endogenously (Tischkau et al., 2003b). This rise in cGMP/activation of PKG is required for clock dynamics to proceed; if either is blocked, clock state reverts by 3.5 h and then recapitulates the intervening period (Tischkau et al., 2004). Peak PKG activity may signal the transition from night to the daytime state. A light-pulse at late subjective night may prematurely shift the clock to this state, thus advancing the clock phase to a point it normally transits at the end of the night state of the circadian cycle and that is required for entry into the daytime state.

### MAPK

Three members of the mitogen-activated protein kinase (MAPK) family, ERK, JNK, and p38, undergo oscillations in phosphorylation in hamsters, with peak phosphorylation levels during the day. All three are phosphorylated in response to light at mid-subjective night (Pizzio et al., 2003). In mice, ERK1 and ERK2 signaling is induced by light pulse in the subjective night. Levels of phosphorylated ERK (pERK) show a circadian oscillation, with peak levels at mid-to late-subjective day (Obrietan et al., 1998).

### CREB

Phosphorylation of the Ca^2+^-cAMP response element-binding protein (forming pCREB) links stimulation of these intracellular signaling pathways to transcriptional activation of genes whose promoters bear the Ca^2+^-cAMP response element (CRE). An endogenous oscillation in basal levels of pCREB occurs in rat and mouse SCN, with a corresponding oscillation in expression of CRE-mediated genes. pCREB levels peak from the middle to end of subjective night. This is followed by peak expression of CRE-mediated genes, which occurs from late-subjective night to mid-subjective day (Obrietan et al., 1999). Light induces ^133^Ser pCREB at levels proportionate to light intensity and expression of CRE-mediated genes only at subjective night (Ginty et al., 1993; Ding et al., 1997; Gau et al., 2002). This indicates that there is strict regulation of CRE-mediated induction of gene expression during the circadian cycle, with only the night being favorable for the CREB/CRE-transcription pathway (Ding et al., 1997).

### BDNF

BDNF mRNA levels in the SCN peak during the early subjective day, and BDNF protein levels peak during the subjective night (Liang et al., 1998). The expression of this rhythm in BDNF is dependent on a tetrodotoxin (TTX)-sensitive neuronal circuit (Baba et al., 2008). These observations support a role for BDNF in gating circadian responses to nocturnal light.

### GABA

There is a diurnal rhythm of activity of GAD, which synthesizes GABA (Aguilar-Roblero et al., 1993). GABA release within the SCN also oscillates (Aton et al., 2006), peaking at the early night (Itri and Colwell, 2003). The peak in GABA release coincides with the timing of the most hyperpolarized state of Vm in SCN neurons (Wang et al., 2012). This release pattern is modulated by VIP signaling (Itri and Colwell, 2003; Itri et al., 2004; Aton et al., 2006).

### Conclusion

Endogenous rhythms in expression/function of these effectors and modulators are timed to allow temporally specific signaling cascades. Functional states of SCN cells with respect to these molecules are varied in *advance* of the point in time when the plasticity-inducing event is anticipated, namely light in the early or late night (Gillette and Wang, 2014). The clock thus *primes* cells to respond to plasticity signals in a specific, time-of-day dependent manner.

These intracellular effectors overlap with some of the effectors that contribute to the standard paradigm for *metaplasticity*, the changes in the state of synapses or neurons that impact the amplitude and persistence of *subsequent* instances of plasticity (Abraham, 2008). Circadian rhythms have been implicated previously as a form of metaplasticty (Gerstner and Yin, 2010; Gerstner, 2012). Therefore, we propose, that the alteration of signal responsivity of SCN cells is a metaplastic form of modulation of plasticity.

With the core clock driving SCN metaplasticity, SCN neurons can show *cyclic* variation in their metaplastic state. This is distinct from metaplastic regulation as it has been described previously (Abraham and Bear, 1996; Abraham, 2008). Persistence of clock-driven metaplasticity is restricted to a regular, discrete period within the 24-h daily cycle. Metaplastic effects that have been studied previously persist from a few minutes to a few days (Abraham, 2008). Therefore, we propose that the repetitive pattern of circadian neuronal state-changes constitutes a paradigm of *iterative metaplasticity* (Figure 1).

## Commonalities in plasticity pathways: sites of clock-driven metaplasticity in the hippocampus

The best-characterized form of activity-dependent synaptic plasticity is long-term potentiation (LTP). Defined as persistent enhancement in synaptic efficacy due to repeated activation of the same synapses, it is widely considered, along with long-term depression (LTD), to be the physiological basis for acquiring new memories and enhancing nascent ones (Malenka and Nicoll, 1999; Lisman, 2003; Cooke and Bliss, 2006). While the molecular basis of LTP is still incompletely determined, the roles of a large number of signaling mechanisms and molecular effectors have been elucidated. Most *in vitro* studies on LTP have been conducted by delivering high frequency stimulation to Schaffer collateral fibers connecting the CA3 and CA1 pyramidal neurons. Glutamatergic synapses were the first investigated for LTP and the well-studied synapses between Schaffer collateral fibers and CA1 pyramidal neurons exhibit LTP mediated by NMDA•R activation (Bliss and Collingridge, 1993; Lisman, 2003). There is, however, synapse-to-synapse heterogeneity in the molecular mechanisms associated with LTP.

Deeper understanding of the signaling mechanisms underlying synaptic plasticity has raised questions as to the manner in which these plasticity mechanisms are modulated. While a number of factors can regulate plasticity at the time of occurrence, activity-dependent processes that modulate plasticity by altering the state of synapses or cells *prior* to plasticity-inducing events are metaplastic. That is, *synaptic metaplasticity* can be considered the *plasticity of synaptic plasticity*. It acts to enhance the salience of subsequent exposure to certain types of stimulation, and prevent saturation of LTP and LTD, which can have negative effects on learning and memory as well as neuronal health (Deisseroth et al., 1995; Philpot et al., 2007; Abraham, 2008; Mockett and Hulme, 2008).

Key to the concept of metaplasticity is that any physiological or biochemical change in the state of the cell or synapse needs to persist beyond the initial activity that triggers the metaplastic changes. This distinguishes the paradigm from direct synaptic regulation, which occurs concurrently with synaptic plasticity. The initial bout of activity, or *priming*, changes the functional state of the synapse, neuron, and network, and thus its susceptibility to future plasticity-inducing events (Abraham, 2008; Hulme et al., 2014).

There is emerging evidence demonstrating that the time-of-day affects the magnitude and persistence of synaptic plasticity. Several studies have shown circadian variation in the efficacy of LTP (Chaudhury et al., 2005; Nakatsuka and Natsume, 2014). Time-of-day effects may be one mechanism of metaplasticity. How the brain clock interacts with synaptic plasticity to cause circadian variation is a question ripe for deeper investigation. Both direct and indirect interactions can be hypothesized.

In this review, we have detailed effectors of SCN plasticity that are under circadian control. Several of these effectors are also involved in hippocampal LTP, including glutamate, NMDA•R, CaMKII, Ca^2+^, NO, RyR, cGMP/PKG, cAMP/PKA, MAPK, and CREB (Lu et al., 1999; Lu and Hawkins, 2002; Monfort et al., 2002; Cooke and Bliss, 2006; Irvine et al., 2006; Zorumski and Izumi, 2012). While a detailed description of the role of these molecules in LTP is outside the scope of this review, the commonality of signaling molecules between plasticity-inducing events in the SCN and hippocampal LTP is striking. Further, signaling cascades involving some of these molecules are also common to both processes. The NO-GC-cGMP-PKG pathway, which is critical in late night signaling in the SCN, has also been shown to contribute to late-phase LTP (L-LTP) (Lu et al., 1999; Lu and Hawkins, 2002; Ping and Schafe, 2010). The cAMP-MAPK-CREB pathway is also involved in both signaling processes (Gerstner and Yin, 2010). In both cases, this signaling cascade induces plasticity by targeting CREB. Glutamate-induced NMDA•R activation, Ca^2+^ influx, and CaMKII phosphorylation are critical signals that mediate both hippocampal LTP and SCN state changes (Malenka and Bear, 2004). Further, several of the signaling elements described, including NMDA•R, Ca^2+^_i_, PKC, NO, CaMKII, and MAPK, previously have been hypothesized as sites for metaplastic regulation of synaptic plasticity (Abraham, 2008; Lucchesi et al., 2011; Zorumski and Izumi, 2012). Lastly, hippocampal plasticity is modulated by molecules that are also modulators of SCN plasticity, like BDNF (Bramham and Messaoudi, 2005; Lu et al., 2008; Minichiello, 2009; Schildt et al., 2013) and GABA (Arima-Yoshida et al., 2011; Nakatsuka and Natsume, 2014).

Cycling of the circadian clock results in iterative metaplasticity via regulation of the effectors and modulators of plasticity in the SCN. Might such a relationship also exist between clock cycling and hippocampal plasticity? One indication of such a paradigm comes from a study that showed that activity of MAPK as well as adenylyl cyclase, and levels of cAMP vary in a circadian fashion in the hippocampus. These oscillations parallel Ras activity and phosphorylation of MAPK kinase and CREB. The variations persist under free-running conditions, indicating they are endogenous in nature. These oscillations were shown to impact long-term memory (Eckel-Mahan et al., 2008). A further study showed that oscillations of adenylyl cyclase and MAPK in the hippocampus are dependent upon an intact SCN (Phan et al., 2011).

A role for GABA in circadian rhythms of LTP has been demonstrated, with nighttime disinhibition of a GABA_A_ network shown to facilitate LTP in the CA1 region of the rat hippocampus (Nakatsuka and Natsume, 2014). Further, the clock gene *Per 2* oscillates in isolated hippocampal slices, indicating the presence of an endogenous clock in the hippocampus. In support of this link, mutations in *Per2* cause abnormal LTP in the hippocampus, mediated by decreased phosphorylation of CREB (Wang et al., 2009).

While the peripheral hippocampal clock may itself drive rhythms in levels/activity of specific molecules, there also may be signals from the core SCN clock that drive or synchronize these rhythms. Signals, such as neuropeptides, hormones, and small molecules, may reach the hippocampus directly, or regulate hippocampal activity indirectly. For instance, signaling from the SCN is known to be critical for rhythmic variation in hormone levels. Several hormones that are known to affect hippocampal LTP, including melatonin and cortisol, are released in circadian patterns controlled by the SCN (Reppert et al., 1981; Gillette and Mitchell, 2002; Chaudhury et al., 2005; Chan and Debono, 2010). This could be one mechanism by which the cycling of the clock can lead to time-of-day changes in LTP. In the SCN itself, glutamate application has been shown to induce LTP of field potentials activated by RHT stimulation in a time-of -day dependent manner. These experiments were performed with SCN slices *in vitro*, indicating that the core clock contributes to these time-of-day dependent changes in LTP (Nisikawa et al., 2002).

We propose that **(1)** the commonality of plasticity elements in the SCN and hippocampus, **(2)** the existence of a circadian clock in the hippocampus that modulates the ability to acquire LTP over ~24-h, and **(3)** evidence for communication between the SCN and the hippocampus all point to clock-driven, iterative metaplasticity in the hippocampus. Further exploration around-the-day of hippocampal expression and activity of molecules, such as NO, PKG, GABA, that have been discussed in this review will shed more light onto clock driven iterative metaplasticity in the hippocampus. The 12-h-limited duration of the persistence of SCN metaplasticity each 24-h cycle makes it a valuable model to probe how such processes affect neurophysiology and the molecular effectors of synaptic plasticity. Such understanding is also critical to elucidating other, non-circadian mechanisms of metaplasticity.

## Conclusion

Considering circadian gating of inputs in the context of hippocampal and SCN metaplasticity provides striking insights on the ways in which signaling mechanisms conserved between two brain regions can impact their functional states. As a well studied system with an iterative impact on plasticity, the circadian clock offers a compelling model system for the study of metaplasticity. Conversely, new insights on links between circadian rhythms and synaptic plasticity can positively impact the study of SCN and hippocampal function. Further understanding of the interactions between these two critical brain processes will require that future research in either field is more deeply informed by the distinct methodological considerations of the other. The potential benefits to understanding the substrates and dynamics of cognitive disorders in both cases can be enormous.

Box 1 **Key Concepts**

**Circadian gating**

Restricted sensitivity and response to plasticity signals that depends upon the time of day. Whether, and to what extent, phase is reset by neurotransmitter signals is under the control of the circadian clock, which is able to gate sensitivity by regulating membrane state and the expression and function of various signaling effectors of plasticity.

**Circadian plasticity**

A persistent change of circadian-clock state in response to significant stimulation during a discrete phase of the ~24-h cycle. When it occurs inappropriately at night, environmental light can permanently change, or reset, the state of the SCN clock. The clock dynamically controls its own susceptibility by circadian plasticity.

**Endogenous circadian rhythms**

Autonomous, self-generated near-24-h rhythms at any level organization of life, such as expression/function of proteins, cellular physiology, neuropeptide release, and amplitude of behavior. Circadian rhythms are defined by their ability to persist with near-24-h periods in the absence of exogenous temporal cues. For example, animals maintained in aperiodic environments such as constant darkness and SCN brain slices maintained *in vitro* exhibit various circadian rhythms, ranging from the rhythmic patterning of wakefulness and sleep, neuronal firing rate, neuropeptide release to cellular metabolic state. They are driven at the cellular level by a transcription-translation oscillator (TTO) and a redox oscillator (RXO).

**Metaplasticity**

A plasticity regulatory phenomenon where experience alters the ability of a system to respond to a subsequent plasticity-inducing stimulus. Metaplasticity was originally described as a mechanism for regulating and tuning synaptic plasticity, but also can alter cell or network state. A critical feature of metaplasticity is that once triggered, metaplastic change must persist long enough to impact a plasticity event occurring at a later time. A metaplastic state can either increase or decrease the amplitude or duration of responses to a later plasticity event.

**Iterative Metaplasticity**

Gating of receptivity to subsequent signals that repeats on a cyclic timebase. An example is gating of susceptibility to light-induced plasticity by the dynamic of the circadian clock in the SCN. Gating of receptivity is achieved by clock-generated, ~24-h rhythms in neuronal membrane state and/or expression or activation state of intracellular signaling pathways permissive for light/glutamate stimulation.

## Author contributions

Rajashekar Iyer, Tongfei Wang and Martha Gillette wrote the text; Martha Gillette revised the text, and provided expertise and funding.

### Conflict of interest statement

The authors declare that the research was conducted in the absence of any commercial or financial relationships that could be construed as a potential conflict of interest.

## Abbreviations

AVP: arginine vasopressin
BDNF: brain-derived neurotrophic factor
CaMKII: calcium-calmodulin dependent protein kinase II
cAMP: cyclic adenosine triphosphate
cGMP: cyclic guanine monophosphate
CICR: calcium-induced calcium release
CREB: cAMP response element-binding protein
DHA: dehydroascorbic acid
ERK: extracellular signal-regulated kinase
GABA: gamma-amino butyric acid
GAD: glutamic acid decarboxylase, GC, guanylate cyclase
GRP: gastrin-releasing peptide
GSH: glutathione
GSSH: glutathione disulphide
ipRGCs: intrinsically photoreceptive retinal ganglion cells
JNK: c-jun kinase
K2P: two-pore domain potassium channel
KCNK: potassium channel subfamily K
LTD: long-term depression
LTP: long-term potentiation
MAPKK/MEK: MAPK kinase
MAPKs: mitogen activated protein kinases
NMDA/ NMDA•R: N-methyl D-aspartate/ N-methyl D-aspartate receptor
NO: nitric oxide
NOS: nitric oxide synthase
NPAS2: neuronal PAS-domain protein 2
ODNs: oligodeoxynucleotides
PACAP: pituitary adenylate cyclase-activating peptide
PKA: cAMP-dependent protein kinase
PKG: cGMP-dependent protein kinase
PVN: paraventricular nucleus
RHT: retinohypothalamic tract
ROR: retinoic acid-related orphan receptor
RXO: reduction-oxidation oscillator
RyR: ryanodine receptor
SCN: suprachiasmatic nucleus
TASK: TWIK-related acid-sensitive K^+^ channel
tPA: tissue-type plasminogen activator
TREK: TWIK-related K^+^ channel
TrkB: tropomyosin-related receptor kinase
TTO: transcription-translation oscillator
TTX: tetrodotoxin
TWIK: Tandem of P domains in a Weak Inward rectifying K^+^ channel
VIP: vasoactive intestinal peptide
VIP•R (also VPAC•R): vasoactive intestinal peptide receptor
V_m_: membrane potential.
